# Understanding the relationship between cerebellar structure and social abilities

**DOI:** 10.1186/s13229-023-00551-8

**Published:** 2023-05-15

**Authors:** Yannis Elandaloussi, Dorothea L. Floris, Pierrick Coupé, Edouard Duchesnay, Angeline Mihailov, Antoine Grigis, Indrit Bègue, Julie Victor, Vincent Frouin, Marion Leboyer, Josselin Houenou, Charles Laidi

**Affiliations:** 1 Sorbonne Université, UFR Médecine, 75005 Paris, France; 2 Department of Adult Psychiatry IMPACT - Mondor University Hospitals AP-HP, Créteil, France; 3 Fondation FondaMental, 94010 Créteil, France; 4 CEA, Neurospin, Université Paris-Saclay, Gif-sur-Yvette, France; 5 Methods of Plasticity Research, Department of Psychology, University of Zurich, Zurich, Switzerland; 6 Donders Institute for Brain, Cognition, and Behavior, Radboud University Nijmegen, Nijmegen, The Netherlands; 7 Department for Cognitive Neuroscience, Radboud University Medical Center Nijmegen, Nijmegen, The Netherlands; 8 Pictura Research Group, Unité Mixte de Recherche Centre National de la Recherche Scientifique (UMR 5800), Laboratoire Bordelais de Recherche en Informatique, Centre National de la Recherche Scientifique, Talence, France; 9 Laboratory for Clinical and Experimental Psychopathology, Department of Psychiatry, University of Geneva, Geneva, Switzerland; 10 University Hospital of Geneva, Geneva, Switzerland; 11 Laboratory of Applied Neuroscience, Department of Psychiatry, Beth Israel Deaconess Medical Center and Harvard Medical School, Boston, USA; 12 Univ Paris Est Créteil, INSERM U955, IMRB, Translational Neuro-Psychiatry, 94010 Créteil, France; 13 Child Mind Institute, Center for the Developing Brain, New York, NY USA

**Keywords:** Cerebellum, MRI, Volumetry, Parcellation, Social communication, Autism

## Abstract

**Background:**

The cerebellum contains more than 50% of all neurons in the brain and is involved in a broad range of cognitive functions, including social communication and social cognition. Inconsistent atypicalities in the cerebellum have been reported in individuals with autism compared to controls suggesting the limits of categorical case control comparisons. Alternatively, investigating how clinical dimensions are related to neuroanatomical features, in line with the Research Domain Criteria approach, might be more relevant. We hypothesized that the volume of the “cognitive” lobules of the cerebellum would be associated with social difficulties.

**Methods:**

We analyzed structural MRI data from a large pediatric and transdiagnostic sample (Healthy Brain Network). We performed cerebellar parcellation with a well-validated automated segmentation pipeline (CERES). We studied how social communication abilities—assessed with the social component of the Social Responsiveness Scale (SRS)—were associated with the cerebellar structure, using linear mixed models and canonical correlation analysis.

**Results:**

In 850 children and teenagers (mean age 10.8 ± 3 years; range 5–18 years), we found a significant association between the cerebellum, IQ and social communication performance in our canonical correlation model.

**Limitations:**

Cerebellar parcellation relies on anatomical boundaries, which does not overlap with functional anatomy. The SRS was originally designed to identify social impairments associated with autism spectrum disorders.

**Conclusion:**

Our results unravel a complex relationship between cerebellar structure, social performance and IQ and provide support for the involvement of the cerebellum in social and cognitive processes.

**Supplementary Information:**

The online version contains supplementary material available at 10.1186/s13229-023-00551-8.

## Background

The cerebellum represents 80% of the surface area of the neocortex and contains more than 50% of all neurons in the brain [1]. While the cerebellum was traditionally known to be involved in motor control, it is now demonstrated—since the seminal work of Jeremy Schmahmann and colleagues [2]—that the posterior portion of this region is implicated in a broad range of cognitive functions.

The posterior cerebellum is involved in social cognition [3] and more specifically in interpreting goal-directed actions through the movements of other persons (“social mirroring”), as well as in social understanding of other individuals’ mental states (“social mentalizing”); see Van Overwalle for a consensus paper on cerebellum and social cognition [4]. The cerebellum is also involved in a broad range of cognitive functions (such as working memory, language processing) [5–7] and general intelligence [8]. Hogan et al. [6] reported that the cerebellar gray matter structure could predict general cognitive ability.

Autism spectrum disorder (hereafter “autism”) is a heterogeneous neurodevelopmental condition affecting 1% of the world population [9] and is characterized by difficulties with social cognition as well as other features such as repetitive and restrictive behaviors. Postmortem studies have found alterations of Purkinje cells in the cerebellum of individuals with autism [10]. Atypicalities in the cerebellum have been found using structural MRI, functional resting state MRI and diffusion MRI [11]. Preclinical models of autism also reported differences in the cerebellum [12, 13] and suggest that the cerebellum might be targeted to rescue behavioral deficits related to autism in mouse models, using Designer Receptors Exclusively Activated by Designer Drugs (DREADD). Cerebellar noninvasive brain stimulation using transcranial magnetic stimulation (TMS) or transcranial direct current stimulation (tDCS) is also under investigation in children with autism (https://clinicaltrials.gov/ct2/show/NCT04446442) as Stoodley et al. [13] found that, in healthy subjects, cerebellar tDCS could modulate the cerebellar functional connectivity that is altered in autism. Based on these studies and their potential therapeutic implication, it is important to understand which regions of the cerebellum might be atypical in autism, and how these regions might be related to the different types of features in autism.

Differences of the posterior–superior cerebellum have been repeatedly reported in autism, in neuroimaging studies with small sample sizes [14]. However, a recent meta-analysis [15] found no evidence of cerebellar alterations in autism. These results are in line with those from our group [16] finding no significant difference in the cerebellar structure between individuals with autism and controls. Different reasons may explain these inconsistent findings, including false positives related to small sample sizes [17] or heterogeneous parcellation methods [14]. The absence of consistent cerebellar atypicalities in autism might be related to the limitations associated with case–control studies in heterogeneous conditions such as autism [18] where graded psychopathological phenotypes may exist. Instead, a dimensional approach in large transdiagnostic populations might unravel more robust brain–behavioral correlations, in line with the Research Domain Criteria approach [19]. Moberget et al. 2019 [20] studied the association between cerebellar structure, psychopathological dimensions, general cognitive function, but not social performance.

We sought to study how social cognition difficulties are related to cerebellar structure in a large transdiagnostic pediatric cohort. More specifically, we hypothesized that the gray matter volume in posterior–superior lobe of the cerebellum (encompassing lobule VI, Crus I, Crus II and lobule VIIb) would be associated with social performance in this population.

## Methods

### Participants

All data analyzed in this study were collected from the Healthy Brain Network (HBN) project [21]. This openly shared dataset is designed to include 10,000 participants. The HBN cohort is a large transdiagnostic dataset of both brain imaging and clinical/behavioral assessments from children and adolescents (5–21 years) with psychiatric disorders or at risk for such disorders [21]. The inclusion criteria of HBN are broad and only require participants to be between 5 and 21 years of age, to speak English and to be able to undergo a clinical evaluation. The exclusion criteria include having a severe neurological disorder or suffering from an acute psychotic episode. Written informed consent was obtained from participants aged 18 years or older, and from legal guardians, in addition to themselves, for those under 18 years old. This protocol was approved by the Chesapeake Institutional Review Board, is conducted following the Declaration of Helsinki for human research and is described elsewhere [21].

We excluded from our study all participants with an IQ below 70 measured with the Wechsler Adult Intelligence Scale (WASI-II) or the Wechsler Intelligence Scale for Children (WISC-V) depending on the age of the participants [22]. We chose to exclude individuals with an IQ below 70 to study a clinical population without intellectual disability, in which it might be difficult to interpret performance in social cognition, as psychometric properties of Social Responsiveness Scale have been validated only in individuals without cognitive impairments [23, 24].

### Phenotyping and clinical assessment

The full clinical assessment of the HBN cohort is described elsewhere [21].

Our goal was to study how social difficulties related to autism were associated with cerebellar anatomy. Social difficulties related to autism were measured with the second version of the Social Responsiveness Scale (SRS) [25]. The SRS-2 is commonly used to measure the severity of autistic symptoms and comes with different versions depending on age. Due to demographic characteristics of the HBN cohort, we used the SRS-2 school-age version, completed by a parent. The SRS-2 can be divided into two scores corresponding to the DSM-5 autism clusters of symptoms: a social communication impairment score (SCI; sum of Social Awareness—AWR, Social Cognition—COG, Social Communication—COM, and Social Motivation—MOT subscales) and a restricted/repetitive behavior score (RRB; Mannerisms subscale) [26, 27]. To differentiate the social component related to autism—which we hypothesized to be related to the posterior cerebellum—from the restrictive/repetitive behaviors component, we studied these two components of the SRS scale separately.

### Parcellation of the cerebellum and quality control

MRI scans were acquired on three distinct sites in New York City: Staten Island, Rutgers University and Cornell Brain Imaging Center. Staten Island images were acquired on a 1.5 T Siemens Avanto (*TR* = 2730 ms, *TE* = 1.64 ms, flip angle = 7°, slice number = 176, voxel dimensions = 1.0 × 1.0 × 1.0 mm^3^). Rutgers University images were acquired on a 3 T Siemens Tim Trio (*TR* = 2500 ms, *TE* = 3.15 ms, flip angle = 8°, slice number = 224, voxel dimensions = 0.8 × 0.8 × 0.8 mm^3^). Cornell Brain Imaging Center images were acquired on a Siemens Prisma 3 T MRI (*TR* = 2500 ms, *TE* = 3.15 ms, flip angle = 8°, slice number = 224, voxel dimensions = 0.8 × 0.8 × 0.8 mm^3^).

**Fig. 1:**
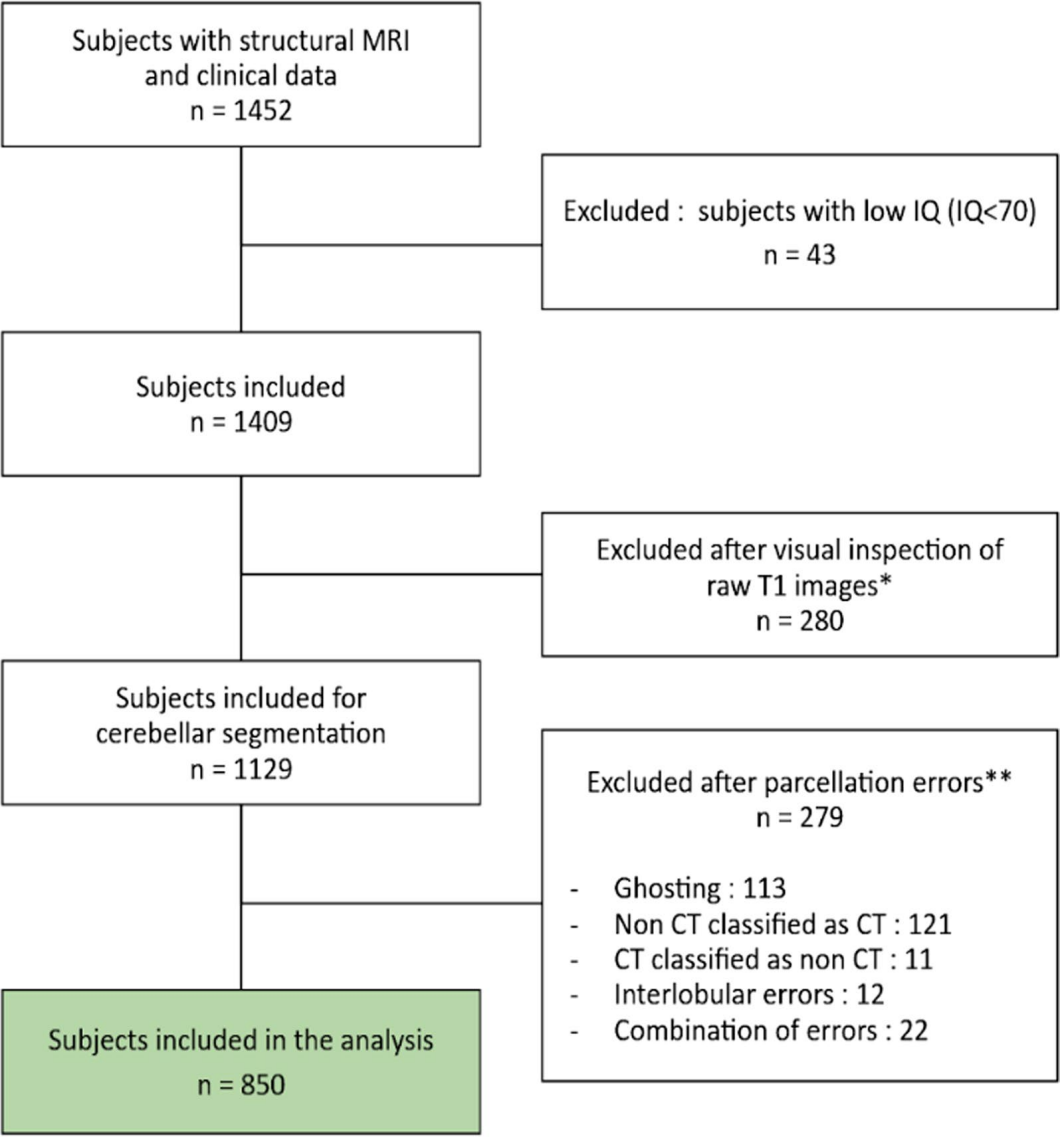
Flowchart diagram. IQ, intelligence quotient; CT, cerebellar tissue. *First quality check procedure: visual inspection of 3 raw T1 slices for each subject. **Second quality check procedure: visual inspection of cerebellar segmentation of T1 images masked with the parcellation outcome for each subject. Examples of excluded subjects are detailed in Additional file 1: Fig. S1

First, we inspected the T1 MRI and excluded those with evident motion from further analyses (see flowchart in Fig. 1). Second, we conducted a fully automated well-validated [28] cerebellar parcellation with the CERES pipeline [29]. All data were processed on a high computing performance cluster in Bordeaux by the team that developed the CERES pipeline (PC). Next, an expert rater (YE)—blind to the clinical features of each participant—visually assessed the quality of MRI scans in every slice for each spatial plan of the cerebellum. We identified subjects with non-cerebellar voxels labeled as voxels belonging to the cerebellum, and vice versa, and subjects with parcellation errors within the cerebellar lobules. The same procedure has been applied previously [16].

All images with parcellation defects were excluded from further analyses (Fig. 1). Illustrations of common cerebellar parcellation errors are reported in Additional file 1: Fig. S1.

### Statistical analyses

All our univariate analyses were conducted with python 3.7 statsmodel open-source library [30], and multivariate analyses with scikit-learn library [31]. Based on the literature [3], we hypothesized that social and communication performance would be associated with atypicalities of the posterior cerebellar gray matter volume, known to be involved in social difficulties. Thus, our main analysis focused on the social communication impairments (SCI) score of the SRS (see phenotypic and clinical assessment section) and its subscales. In secondary analyses, we studied the repetitive and restricted behaviors (RRB) score of the SRS as well as the total SRS score.

### Linear mixed models

We used two linear mixed models to predict cerebellar morphometric features. In the first model, we included SRS-SCI, full-scale IQ (FSIQ), age and total intracranial volume (ICV) as continuous covariates; sex as a categorical covariate and site of MRI acquisition as a random effect. Complex interactions between the diagnosis or the severity of autism and (i) sex [32, 33], (ii) age [34, 35], (iii) IQ [36] or (iv) intracranial volume [37] have been reported in autism. To take into account these interactions, we included four 2-way interactions terms between (i) SRS-SCI and (ii) sex/age/IQ/intracranial volume in a second model as described in Traut et al. paper investigating cerebellar anatomy in autism [15]. Next, we tested if the results of the second model would be altered by collinearity, using the variance inflation factor [38].

**Fig. 2:**
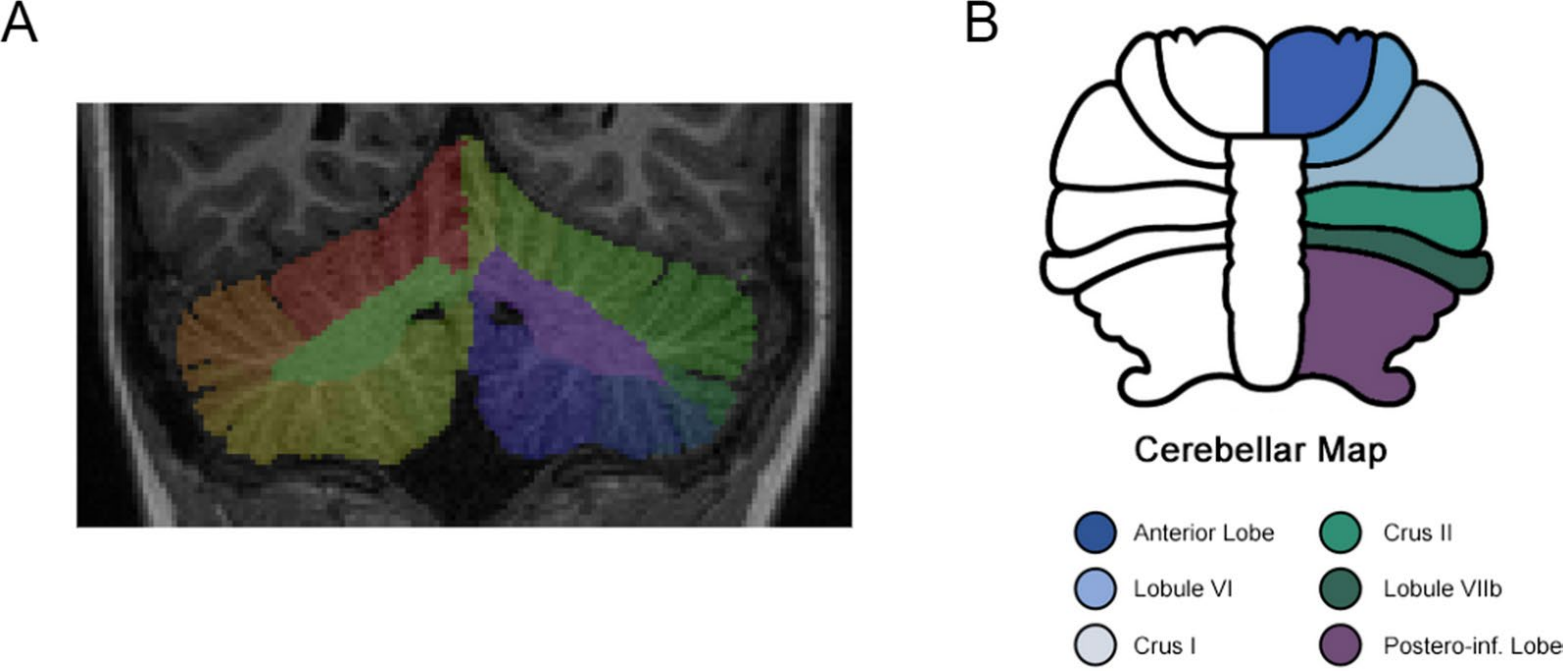
Cerebellar segmentation with CERES and ROI map. **A** Mask of a CERES segmentation output on the native T1 weighted scan. **B** Cerebellar map of lobules and ROI used in linear models. Anterior lobe includes lobules I to V, postero-inferior lobe includes lobules IX and X. Vermis is not segmented by CERES atlas and therefore not colored on the figure

We ensured that the standardized residuals were normally distributed, as assessed by visualization of residuals distribution and QQ-plots. To avoid bias related to outliers and to fulfill linear model assumptions, we excluded observations in each model if studentized residuals were > 3 standard deviations. We ran our analysis with and without outliers to ensure the robustness of our results. We applied a false discovery rate correction (FDR—Benjamini–Hochberg correction) to control for multiple testing (twelve tests, one for each region of interest) (Fig. 2). We present both uncorrected and corrected p values. We report only results surviving significance threshold (*p* < 0.05) after correction for multiple comparisons.

### Canonical correlation analysis

We conducted canonical correlation analysis (CCA) to identify latent dimensions underlying the association between SRS-SCI and cerebellar anatomy. The aim of this analysis was to identify from a multivariate perspective the association between cerebellar anatomy and clinical features of interest. We defined a component with anatomical features “X” and a component with clinical features of interest “Y.” We included in the X component the gray matter volumes of cerebellar parcellation for the left and right cerebellum (11 regions of interest: lobules I-II, III, IV, V, VIIb, VIIIa, VIIIb, IX, X, Crus I, Crus II), the total cerebellum and the intracranial volume, after regressing out the effect of site, age and sex. We included in the Y component the subscales of the SRS-SCI scale and FSIQ after regressing the effect of site, age and sex. We then computed the correlation between the two canonical components for clinical and anatomical features. To assess the significance of our model, we conducted a 10,000-permutation test to define the threshold of significance of our model (*p* = 0.001).

## Results

### Population of the study

From 1452 participants with MRI and clinical data available, we included 850 subjects in our analysis after excluding subjects with low IQ, excessive motion and parcellation errors (see methods section and Fig. 1). Comparisons of the subjects included and excluded from the analyses after the quality control are reported in Additional file 1: Table S1. Subjects excluded from the analyses after quality control were younger, had more severe autistic symptoms and were more likely to be male. This result was expected since younger subjects are more prone to move their head during scanning acquisition. In our final sample, the mean age was 10.75 years old. A description of phenotypic variables is reported in Table 1, and the distribution of main clinical variables is represented in Additional file 1: Fig. S2.

**Table 1:** Demographic characteristics

	Study sample (*N* = 850)
Age	
Mean (SD)	10.8 (3.0)
Min, Max	5.8, 17.9
Sex	
Male	496 (58%)
Female	354 (42%)
IQ Total	
Mean (SD)	99.7 (15.7)
Min, Max	70, 147
SCI	
Mean (SD)	43.0 (23.6)
Min, Max	0, 115
RRB	
Mean (SD)	8.5 (7.1)
Min, Max	0, 32
Total SRS	
Mean (SD)	51.5 (29.8)
Min, Max	0, 147
Diagnosis	
ADHD	357 (42.0%)
Anxiety Disorder	107 (12.5%)
Specific Learning Disorder	71 (8.3%)
ASD	52 (6.1%)
Depressive Disorders	51 (6.0%)
Communication Disorder	16 (1.8%)
Motor Disorder	8 (0.9%)
Other	70 (8.2%)
No diagnosis	117 (12.8%)
Parental Level of Education	
Father	
Less than high school	31 (3.6%)
High school graduate	195 (22.9%)
College education	281 (33.1%)
Graduate degree	329 (38.7%)
No data	14 (1.6%)
Mother	
Less than high school	40 (4.7%)
High school graduate	250 (29.4%)
College education	207 (24.4%)
Graduate degree	213 (25.1%)
No data	140 (13.5%)
Household Income	
Less than $10,000	14 (1.6%)
$10,000 to $49,999	116 (13.6%)
$50,000 to $99,999	150 (17.6%)
$100,000 to $149,999	128 (15.1%)
$150,000 or more	185 (21.8%)
No data	257 (30.2%)

SD, standard deviation; SRS, Social Responsiveness Scale total score; SCI, social and communication SRS’s subscale; RRB, restricted and repetitive behavior SRS’s subscale; IQ, intelligence quotient; ADHD, attention deficit hyperactivity disorder; and ASD, autism spectrum disorder. Percentages may not add up to 100 due to rounding

### Linear models

When conducting linear models without interactions, we found no significant association between any cerebellar volume and SRS-SCI. When including all interactions, the volume of lobule VI (corrected *p* value = 0.028) and Crus II (corrected *p* value = 0.028) was positively associated with the SRS-SCI scale. In addition, the total volume of the cerebellum was also positively associated with the SRS-SCI scale (corrected *p* value = 0.024). Results are reported in Additional file 1: Table S2. As reported in Additional file 1: Table S2, there was a small number of outliers in our linear models, representing < 1% of the total number of subjects. However, when testing for possible overfitting in such models, we found that significant interactions showed a strong collinearity with SRS-SCI scale as assessed by variance inflation factor, suggesting that linear models with multiple interactions were not appropriate.

### Canonical correlation analysis

**Fig. 3:**
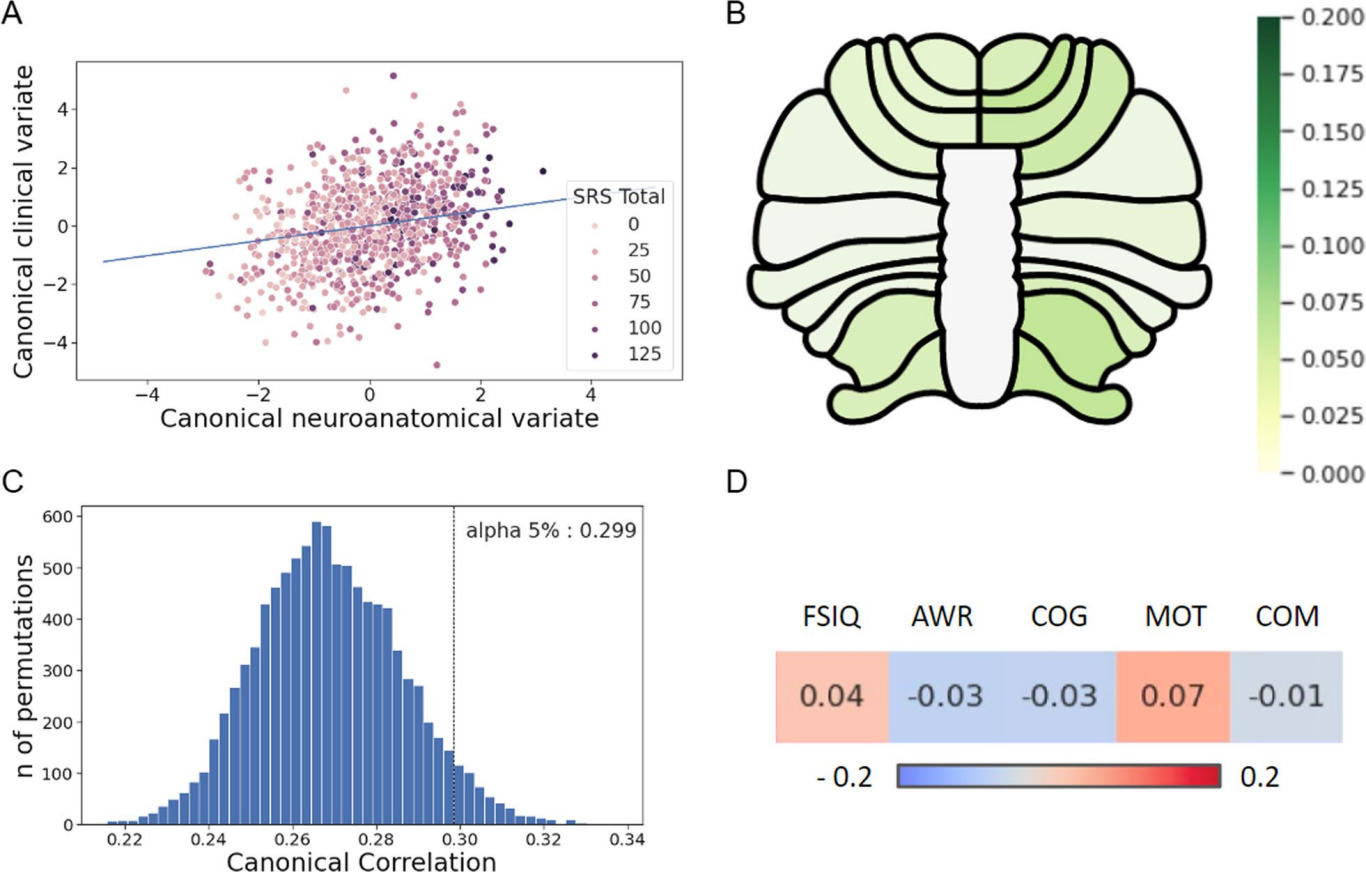
CCA results and significance threshold. **A** Canonical correlation plot between cerebellar domain and clinical domain. **B** Cerebellar map showing correlation coefficients between each lobule and canonical clinical variate. **C** Distribution of canonical correlation coefficients between clinical and neuroanatomical domains when performing shuffle (10,000 permutations) and significance threshold for an alpha level of 0.05. Our model was significant at *p* < 0.0001 threshold (*r* = 0.36). **D** Correlation coefficients between each clinical variable and canonical neuroanatomical variate. CCA, Canonical Correlation Analysis; SRS, Social Responsiveness Scale total score; FSIQ, Full-Scale Intelligence Quotient; AWR, SRS Social Awareness subscale; COG, SRS Social Cognition subscale; MOT, SRS Social Motivation subscale; and COM, SRS Social Communication subscale

In the CCA analysis, we found a significant correlation between the canonical clinical variate and the canonical neuroanatomical variate (*r* = 0.36; *p* < 0.0001). Results are reported in Fig. 3. The Social Motivation variable and FSIQ were the two main clinical variables associated with the canonical clinical variate (see Additional file 1: Fig. S3). To test the specificity of the association between social communication impairment and cerebellar structure, we repeated the CCA analyses excluding FSIQ. In that case, the correlation between the clinical (SRS subscales) and the anatomical component was not significant.

### Secondary analyses

Results from the CCA underlined the relevance of combining FSIQ and SRS subscales in the clinical component of our analysis. We tested the effect of FSIQ on cerebellar volume using linear models, considering sex, age, ICV and site of inclusion as covariates. We found no statistically significant effect of FSIQ on any of the cerebellar lobules. In addition, we tested the correlation between the SRS-SCI score and FSIQ and found a strong association between both measures (*r* = 0.24, *p* < 0.001). We did not find any significant association between cerebellar lobules and the RRB scale. In addition, analysis of cerebellar lobules and total score of the SRS scale did not reveal any statistically significant association after correction for multiple testing. Results are reported in Additional file 1: Table S3. Last, we found no statistical association between any of the SRS-SCI subscores (AWR, SOC, MOT, COM) and cerebellar structure in linear models.

## Discussion

Our goal was to investigate the association between social communication performance and the cerebellar morphology in a large multicenter transdiagnostic pediatric cohort. We did not identify significant associations with social communication performance using linear mixed models. However, in the CCA analysis, we found a significant association between the canonical clinical variate (including SRS-SCI subscales and FSIQ) and the canonical cerebellar neuroanatomical variate.

Our results suggest that the cerebellar structure is associated with social cognition and FSIQ, but that there is no one-to-one relationship between one lobule of the cerebellum and social skills, but rather a “many-to-many” relationship that can be more accurately captured by CCA models [39]. This adds to a growing body of the literature on the role of the cerebellum in social cognition and general cognitive abilities. Neurodevelopmental alterations in the cerebellar structure could lead to atypicalities in multiple cognitive domains and disrupt cerebello-thalamo-cortical pathways. Indeed, Wang et al. [40] suggest that an insult in the cerebellum during sensitive periods of brain development might be causal and have a distant effect on cortical structures.

The postero-superior “cognitive” cerebellum has been implicated in several psychiatric disorders. For example, there is now strong evidence [20, 41, 42] that the volume of the posterior cerebellum is reduced in schizophrenia. In the field of autism, despite initial studies in small samples [11, 43] suggesting that there might be atypicalities in this region, recent larger studies [15, 16] found no compelling evidence for volumetric alterations of the cerebellum. These studies typically rely on case/control comparisons. However, autism is a heterogeneous construct with many co-occurring conditions such as ADHD, mood disorders, anxiety or learning disabilities [44]. These factors might explain why to date case–control studies have failed to identify reliable biomarkers in autism.

To overcome these limits, a dimensional approach is key to better understand the relationship between the brain anatomy and symptoms of neurodevelopmental disorders. The HBN cohort is a transdiagnostic pediatric cohort that allows this type of analysis [21]. The inclusion criteria are very broad: Approximately half of the participants are suffering from a neurodevelopmental disorder, 20% from anxiety or depression and 10% did not have a diagnosis after the clinical evaluation. Our goal was to understand, in this pediatric population, how social communication performance might be related to cerebellar structure.

We tested two linear models for each region of interest. In the first one, we considered age, sex, site and ICV as covariates/cofactors, and in the second one, we added in addition interactions between the social communication performance and sex, age, ICV and IQ. While the model without interactions revealed no significant results, we found significant results in the second model. However, the significant interaction terms varied depending on the region of the cerebellum, suggesting that a single model would not have been appropriate for all regions of interest. Lastly, we found high collinearity bias, underlining the limits of linear mixed models to study brain–behavior association. To overcome this limitation, we conducted a canonical correlation analysis to study the association between cerebellar neuroanatomy and social communication performance in a multivariate fashion with a CCA model [39]. Previous studies used CCA models to investigate complex relationships between imaging data and cognitive variables [45, 46]. Our goal was to explore in a single analysis the complex relationship between clinical and anatomical features. The CCA model allowed us to include both SRS social subscales and FSIQ in the canonical clinical variate, instead of treating FSIQ as a confound. We found a significant canonical correlation between the canonical clinical variate (including SRS-SCI subscales and FSIQ) and the canonical cerebellar neuroanatomical variate. The canonical clinical variate loadings revealed a predominant effect of the SRS social motivation subscale and the FSIQ. Autism and intellectual disability are frequently associated conditions [9]. Mice models of autism, such as SHANK or CNTNAP2, are strongly associated with intellectual disability [47]. Following the theory of Wang [40], an insult in the cerebellum during brain development could have consequences on multiple clinical dimensions, such as cognitive functions and social skills.

Our study has several strengths. First, we tested a specific hypothesis in a large multicentric and transdiagnostic pediatric cohort and found that linear models might not be ideal to capture the association between cerebellar anatomy and social communication performance. Instead, CCA allowed us to unravel a more complex association between social communication, FSIQ and the cerebellar structure. Our approach might be relevant to explore the association between other specific brain circuits or regions and clinical features, and not only in brain wide association studies [48]. Second, because we decided to focus on a specific brain region, we were able to perform a careful visual inspection to ensure the quality of the cerebellar parcellation. We decided to perform the cerebellar parcellation with the CERES pipeline. This automated parcellation method is well validated, has been successfully applied to healthy populations [49] and psychiatric disorders [16, 41] and outperforms other cerebellar parcellation methods [28]. To the best of our knowledge, this study is to date the largest to investigate the association between the cerebellar morphology and symptoms related to autism. Third, we performed a thorough and careful visual inspection of all parcellation outcomes and T1 data. After quality check, we excluded 559 images based on motion artifacts movements (n = 280) or parcellation errors (n = 279). This suggests the importance of performing a visual quality inspection of all data, even in large datasets. Given that excluded subjects tend to be younger males and with a slight increase in symptoms compared to other subjects, not performing a careful quality check could lead to spurious results.

Our work opens several perspectives. Our results were the results of an analysis in the Healthy Brain Network [21], a large transdiagnostic developmental cohort. By using a dimensional approach, we were able to go beyond the case–control comparison [16] that might not be the only option to investigate the biological basis of social atypicalities. Our study focuses on structural MRI. However, studying the functional connectivity of the regions of the posterior cerebellum might help to gain more insight on how the cerebellum is interacting with the cerebrum in social cognition. However, one should be cautious on how to interpret the functional connectivity findings, since functional connectivity analyses and the common MNI template rely on the assumption that there are no strong differences in the structural morphology. Atypicalities in cerebellar functional connectivity might be only related to changes in the structural anatomy of this region. Stoodley et al. [13] studied the functional connectivity of the cerebellum in animal models and humans, showing modifications of cerebellar–cerebral cortex connectivity when delivering transcranial direct current stimulation (tDCS) of lobule VII and suggesting a clinical improvement of social abilities after such a stimulation. Defining specific cerebellar regions altered in social and communication symptoms may therefore lead to new therapeutic strategies focused on accurate transcranial stimulation.

## Limitations

Several limits should be considered before interpreting our results. First, we decided to perform cerebellar parcellation and to measure the volume of cerebellar lobules considering our interest from a topographical anatomical perspective. Other methods, such as voxel-based-morphometry (VBM) [50, 51], may be more suitable to capture anatomical patterns at a voxel-level, beyond the anatomical boundaries defined by cerebellar parcellation. Therefore, our method of parcellation may miss anatomical changes independently from lobules boundaries, since the anatomical and functional landmarks of the cerebellum do not overlap [52]. However, a visual quality check can only be performed with cerebellar parcellation, since the subjects are visualized in their own native space. This is not possible in VBM analyses, where subjects are analyzed in a common space. We excluded 40% subjects after our quality assessment, suggesting that this step is critical. In addition, we analyzed a pediatric sample and the cerebellar VBM tools have not been optimized for this age range. Thus, performing a quality assessment in the subject space on parcellated data was important to ensure the robustness of our results. Second, we analyzed the SRS-2 scale to measure social atypicalities, a validated scale for autism symptoms’ screening but that has not been formally validated to screen social atypicalities in other psychiatric disorders. Because our goal was to conduct a transdiagnostic analysis and that the Healthy Brain Network cohort [21] is not designed to conduct case–control analyses, we did not investigate the influence of each diagnosis on the cerebellar anatomy. However, the SRS-2 scale followed a Gaussian distribution in our sample even though only 6% of the individuals had a formal diagnosis of autism, suggesting that the symptoms measured by the SRS were transdiagnostic. In addition, the SRS scale has been used to measure autistic traits in other clinical populations such as eating disorders [53], psychosis [54] or ADHD [55].

## Conclusion

To conclude, we found a canonical correlation between social communication performance and cerebellar neuroanatomy. Our work suggests the interest of CCA models to investigate the link between cerebellar structure and dimensions of psychopathology and adds to the body of the literature on the role of the cerebellum in social and general cognitive functions from a dimensional perspective.

## Supplementary Information

**Additional file 1: Fig. S1** Illustrations of common parcellation errors and motion artifacts. **Fig. S2** Distribution of clinical variables. **Fig. S3** Heatmap of correlations between clinical canonical component, anatomical canonical component and individual variables. **Table S1** Comparison of subjects included and excluded from the analyses after quality control. **Table S2** Association between volumes of the cerebellum and social communication impairment scale, age, sex, IQ, intracranial volumeand interactions between SCI and covariates. **Table S3** Association between volumes of the cerebellum and SRS Total and RRB.

## Data Availability

Data are available on request from the authors.

## Abbreviations

ADHD: Attention deficit hyperactivity disorder
AWR: Social Awareness
CCA: Canonical correlation analysis
CERES: CEREbellum Segmentation
COG: Social Cognition
COM: Social Communication
DREADD: Designer Receptors Exclusively Activated by Designer Drugs
DSM-5: Diagnostic and Statistical Manual of Mental Disorders, Fifth Edition
FDR: False discovery rate
FSIQ: Full‑scale intelligence quotient
HBN: Healthy Brain Network
ICV: Intracranial volume
ID: Intellectual disability
IQ: Intelligence quotient
MOT: Social Motivation
MRI: Magnetic resonance imaging
RRB: Restricted and repetitive behaviors
SCI: Social communication impairment score
SD: Standard deviation
SRS: Social Responsiveness Scale, 2nd Edition
tDCS: Transcranial direct current stimulation
TMS: Transcranial magnetic stimulation
VBM: Voxel‑based morphometry
WASI-II: Wechsler Adult Intelligence Scale, 2nd Edition
WISC-V: Wechsler Intelligence Scale for Children, 5th Edition
