# Correction: Understanding the relationship between cerebellar structure and social abilities

**DOI:** 10.1186/s13229-023-00553-6

**Published:** 2023-07-05

**Authors:** Yannis Elandaloussi, Dorothea L. Floris, Pierrick Coupé, Edouard Duchesnay, Angeline Mihailov, Antoine Grigis, Indrit Bègue, Julie Victor, Vincent Frouin, Marion Leboyer, Josselin Houenou, Charles Laidi

**Affiliations:** 1 Sorbonne Université, UFR Médecine, 75005 Paris, France; 2 Department of Adult Psychiatry IMPACT-Mondor University Hospitals AP-HP, Créteil, France; 3 Fondation FondaMental, 94010 Créteil, France; 4 CEA, Neurospin, Université Paris-Saclay, Gif‑sur‑Yvette, France; 5 Methods of Plasticity Research, Department of Psychology, University of Zurich, Zurich, Switzerland; 6 Donders Institute for Brain, Cognition, and Behavior, Radboud University Nijmegen, Nijmegen, The Netherlands; 7 Department for Cognitive Neuroscience, Radboud University Medical Center Nijmegen, Nijmegen, The Netherlands; 8 Pictura Research Group, Unité Mixte de Recherche Centre National de La Recherche Scientifique (UMR 5800), Laboratoire Bordelais de Recherche en Informatique, Centre National de La Recherche Scientifique, Talence, France; 9 Laboratory for Clinical and Experimental Psychopathology, Department of Psychiatry, University of Geneva, Geneva, Switzerland; 10 University Hospital of Geneva, Geneva, Switzerland; 11 Laboratory of Applied Neuroscience, Department of Psychiatry, Beth Israel Deaconess Medical Center and Harvard Medical School, Boston, USA; 12 Univ Paris Est Créteil, INSERM U955, IMRB, Translational Neuro-Psychiatry, 94010 Créteil, France; 13 Child Mind Institute, Center for the Developing Brain, New York, NY USA

**Correction : Molecular Autism (2023) 14:18** https://doi.org/10.1186/s13229-023-00551-8

Following publication of the original article [1], the authors identified errors in the affiliation: Affiliation 2 was presented incorrectly and affiliation 14 was incorrectly captured as affiliation and should have been removed.

This error is corrected in the affiliations list of this Correction article and the original article [1] has been updated.
