# Supplementary material for: Understanding the relationship between cerebellar structure and social abilities
Source: Mol Autism. 2023 May 15;14:18. doi: 10.1186/s13229-023-00551-8 (PMC10186758; doi:10.1186/s13229-023-00551-8)
Supplement: Supplementary file 1 — Additional file 1: Fig. S1 Illustrations of common parcellation errors and motion artifacts. Fig. S2 Distribution of clinical variables. Fig. S3 Heatmap of correlations between clinical canonical component, anatomical canonical component and individual variables. Table S1 Comparison of subjects included and excluded from the analyses after quality control. Table S2 Association between volumes of the cerebellum and social communication impairment scale, age, sex, IQ, intracranial volumeand interactions between SCI and covariates. Table S3 Association between volumes of the cerebellum and SRS Total and RRB. [file 13229_2023_551_MOESM1_ESM.pdf]

SUPPLEMENTARY MATERIAL

Supplementary Figure 1. Illustrations of common parcellation errors and motion artifacts

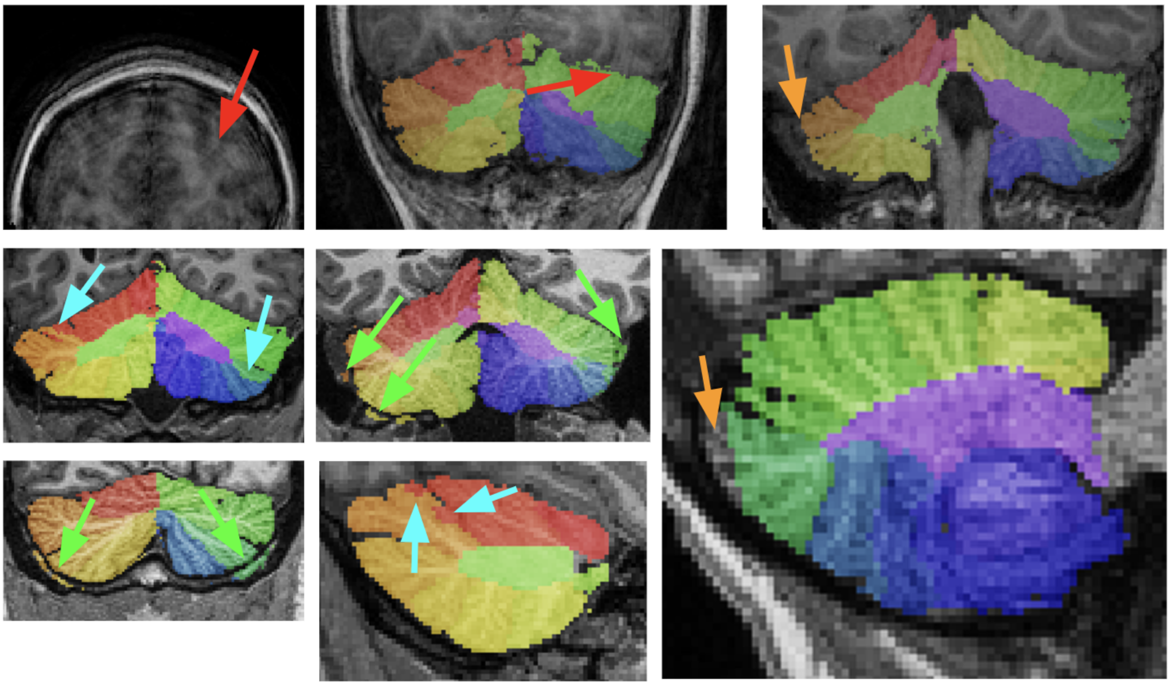

Red arrows: motion related artifacts (ghosting); Orange: Non segmented cerebellar tissue; Cyan: Parcellation errors ; Green : non cerebellar tissue labeled as cerebellar tissue

Supplementary Figure 2. Distribution of clinical variables

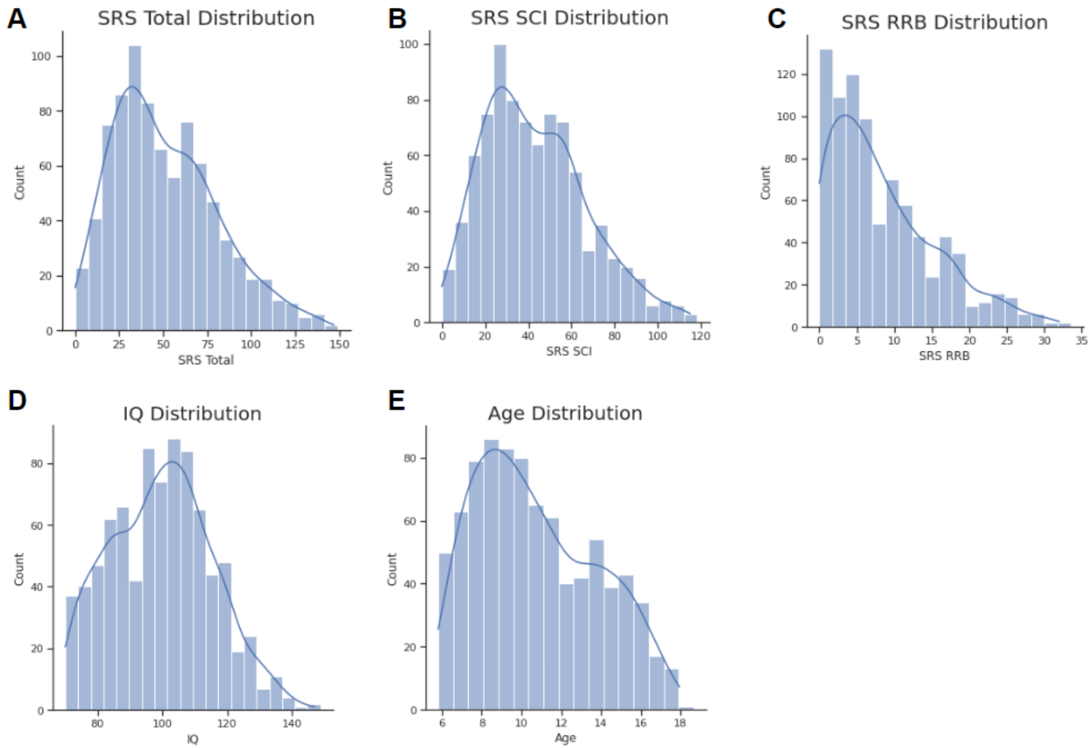

SRS; Social Responsiveness Scale; SCI, social and communication SRS's subscale; RRB, RRB, repetitive behavior SRS's subscale; IQ, Intelligence Quotient.

Supplementary Figure 3. Heatmap of correlations between clinical canonical component, anatomical canonical component and individual variables.

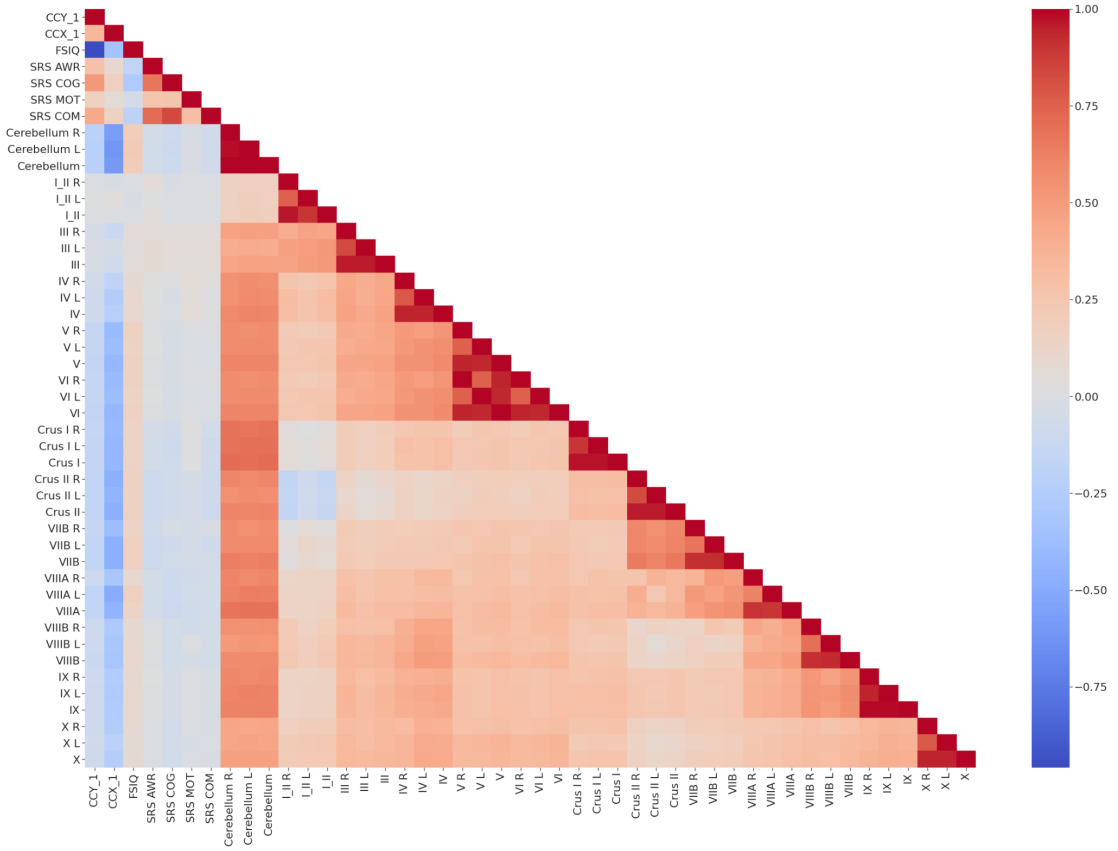

CCY1, Clinical Canonical Variable; CCX1, Anatomical Canonical Variable; FSIQ, Full-Scale Intelligence Quotient; SRS AWR, Social Awareness SRS's subscale; SRS COG, Social Cognition SRS's subscale; SRS COM, Social Communication SRS's subscale; SRS MOT, Social Motivation SRS's subscale

Supplementary Table 1. Comparison of subjects included and excluded from the analyses after quality control

Demographics	Included (N = 850)	Excluded (N = 559)	Statistics	P-val
Mean Age (SD)***	10.8 (3.0)	10.0 (3.1)	Student Z = -4.7	< 0.0001***
Sex (%of M)***	354/496 (58%)	164/395 (71%)	Chi2 T = 21.5	< 0.0001***
SRS Total (SD)*	51.5 (29.8)	55.1 (31.3)	Student Z = 2.1	0.037*
SCI (SD)	43.0 (23.6)	45.6 (24.7)	Student Z = 1.9	0.061
RRB (SD)*	8.5 (7.1)	9.5 (7.5)	Student Z = 2.5	0.011*
FSIQ (SD)	99.7 (15.7)	99.2 (15.0)	Student Z = -1.0	0.328

SD, standard deviation; M, male; SRS Total, Social Responsiveness Scale total score; SCI, social and communication SRS's subscale; RRB, RRB, repetitive behavior SRS's subscale; IQ Total, Intelligence Quotient; significant differences are marked by P-val threshold: < 0.05 (*) or < 0.001 (***).

Supplementary Table 2. Association between volumes of the cerebellum and social communication impairment scale (SCI), age, sex, IQ, intracranial volume (ICV) and interactions between SCI and covariates.

LOBULE	SCI p-value (p-value corr)	SCI coef [95 IC]	AGE p-value	AGE coef [95 IC]	SEX p-value	SEX coef [95 IC]	IQ p-value	IQ coef [95 IC]	ICV p-value	ICV coef [95 IC]	SCI*AGE p-value	SCI*AGE coef [95 IC]	SCI*SEX p-value	SCI*SEX coef [95 IC]	SCI*IQ p-value	SCI*IQ coef [95 IC]	SCI*ICV p-value	SCI*ICV coef [95 IC]	Out.
Cerebellum	0.0034 (0.0239*)	0.547 [0.181; 0.914]	0.0064	0.612 [0.145; 1.078]	0.0269	-3.204 [-6.402; -0.006]	0.0804	0.086 [-0.006; 0.178]	<0.0001	0.074 [0.062; 0.086]	0.0767	-0.008 [-0.017; 0.002]	0.4276	0.018 [-0.047; 0.082]	0.0766	-0.002 [-0.003; 0.000]	0.1177	-0.000 [-0.000; 0.000]	4
Lobe Ant	0.1059 (0.1483)	0.051 [-0.011; 0.113]	0.0339	0.087 [0.008; 0.166]	0.0575	-0.577 [-1.128; -0.026]	0.2445	0.012 [-0.003; 0.028]	<0.0001	0.008 [0.006; 0.010]	0.1576	-0.001 [-0.003; 0.000]	0.5236	0.004 [-0.007; 0.015]	0.2706	-0.000 [-0.001; 0.000]	0.4082	-0.000 [-0.000; 0.000]	3
Lobule VI	0.0117 (0.0280*)	0.104 [0.023; 0.185]	0.4769	0.039 [-0.065; 0.142]	0.8570	0.129 [-0.592; 0.850]	0.6843	-0.001 [-0.021; 0.020]	<0.0001	0.012 [0.009; 0.015]	0.6060	-0.001 [-0.003; 0.002]	0.9638	-0.001 [-0.016; 0.013]	0.9979	-0.000 [-0.000; 0.000]	0.0194	-0.000 [-0.000; 0.000]	2
Crus I	0.1737 (0.1736)	0.092 [-0.041; 0.225]	0.6614	0.011 [-0.159; 0.181]	0.0965	-0.621 [-1.799; 0.557]	0.3029	0.015 [-0.018; 0.049]	<0.0001	0.016 [0.011; 0.020]	0.7069	-0.000 [-0.004; 0.003]	0.3953	0.004 [-0.020; 0.028]	0.3970	-0.000 [-0.001; 0.000]	0.6269	-0.000 [-0.000; 0.000]	2
Crus II	0.0120 (0.0280*)	0.120 [0.026; 0.214]	0.1955	0.059 [-0.062; 0.180]	0.5929	-0.059 [-0.892; 0.775]	0.0204	0.026 [0.002; 0.050]	<0.0001	0.010 [0.006; 0.013]	0.1692	-0.001 [-0.004; 0.001]	0.5249	-0.009 [-0.026; 0.008]	0.0777	-0.000 [-0.001; 0.000]	0.2762	-0.000 [-0.000; 0.000]	5
Lobule VIIB	0.0736 (0.1289)	0.027 [-0.004; 0.093]	0.3418	0.027 [-0.035; 0.089]	0.0718	-0.418 [-0.849; 0.013]	0.0038	0.018 [0.006; 0.030]	<0.0001	0.005 [0.003; 0.006]	0.5662	-0.000 [-0.002; 0.001]	0.1883	0.006 [-0.003; 0.015]	0.0370	-0.000 [-0.001; 0.000]	0.4869	-0.000 [-0.000; 0.000]	5
Lobe Post-Inf	0.1451 (0.1693)	0.088 [-0.031; 0.207]	0.0039	0.228 [0.076; 0.380]	0.0017	-1.789 [-2.848; -0.731]	0.1538	0.022 [-0.008; 0.052]	<0.0001	0.015 [0.011; 0.019]	0.0218	-0.004 [-0.007;-0.001]	0.2926	0.013 [-0.009; 0.034]	0.1128	-0.000 [-0.001; 0.000]	0.7459	-0.000 [-0.000; 0.000]	1

P-value for each variate and interaction terms (P-value after Benjamini-Hochberg FDR correction is added between brackets for the SCI variable).

SCI, social and communication SRS's subscale; IQ, Intelligence Quotient; ICV, intracranial volume. CI95%, Confidence Interval

Supplementary Table 3. Association between volumes of the cerebellum and SRS Total and RRB.

LOBULE	SRS Total		RRB	
	P-value	Corrected P-value	P-value	Corrected P-value
Cerebellum	0.0105	0.0732	0.3236	0.6789
Lobe Ant	0.3593	0.3593	0.5819	0.6789
Lobule VI	0.0247	0.0863	0.5775	0.6789
Crus I	0.2553	0.2979	0.8342	0.8342
Crus II	0.0422	0.0986	0.1325	0.6789
Lobule VIIB	0.1014	0.1775	0.4276	0.6789
Lobe Post-Inf	0.1904	0.2665	0.5731	0.6789

SRS Total, Social Responsiveness Scale total score; RRB, RRB, repetitive behavior SRS's subscale; Benjamini-Hochberg FDR correction.
